# Advances in Microfluidic Single-Cell RNA Sequencing and Spatial Transcriptomics

**DOI:** 10.3390/mi16040426

**Published:** 2025-04-02

**Authors:** Yueqiu Sun, Nianzuo Yu, Junhu Zhang, Bai Yang

**Affiliations:** 1 State Key Laboratory of Supramolecular Structure and Materials, College of Chemistry, Jilin University, Changchun 130000, China; 2 Joint Laboratory of Opto-Functional Theranostics in Medicine and Chemistry, The First Hospital of Jilin University, Jilin University, Changchun 130000, China

**Keywords:** microfluidics, single cell, single-cell RNA sequencing (scRNA-seq), spatial transcriptome

## Abstract

The development of micro- and nano-fabrication technologies has greatly advanced single-cell and spatial omics technologies. With the advantages of integration and compartmentalization, microfluidic chips are capable of generating high-throughput parallel reaction systems for single-cell screening and analysis. As omics technologies improve, microfluidic chips can now integrate promising transcriptomics technologies, providing new insights from molecular characterization for tissue gene expression profiles and further revealing the static and even dynamic processes of tissues in homeostasis and disease. Here, we survey the current landscape of microfluidic methods in the field of single-cell and spatial multi-omics, as well as assessing their relative advantages and limitations. We highlight how microfluidics has been adapted and improved to provide new insights into multi-omics over the past decade. Last, we emphasize the contributions of microfluidic-based omics methods in development, neuroscience, and disease mechanisms, as well as further revealing some perspectives for technological advances in translational and clinical medicine.

## 1. Introduction

Tissue is defined as a unit composed of cells and varying amounts of extracellular matrix, which is the structural and functional part of an organ [1]. Therefore, understanding the tissue microenvironment is the key to unraveling the complex flow of genetic information in living systems. In eukaryotes, revealing the high degree of tissue heterogeneity, both cellular and spatial, in physiological and biochemical phenomena is being increasingly regarded as essential for exploring developmental and disease mechanisms. Various single-cell sequencing (SCS) technologies enable the acquisition of gene expression data at the single-cell level, designed to increase the understanding of cellular diversity and interactions in a physiological and specifically immunological context [2,3,4,5,6,7]. Spatial transcriptome technologies are capable of distinguishing the gene expression of cells in their original locations, thus compensating for the loss of spatial location information during tissue dissociation in SCS technologies [8,9,10]. Notably, the requirements for resolution, throughput, and multiplexing have strongly motivated the development of new solutions for both single-cell and spatial transcriptome sequencing, aiming to generate more detailed molecular cellular landscapes in tissues to unravel cell diversity and explore tissue architectures [11,12,13].

Microfluidics, a tool that integrates microfabrication processes such as microchannels and microvalves, enables the processing of individual cells and their components through the precise control and manipulation of microscale fluids [14]. In the last decade, microfluidics has redefined the experimental platform for SCS and spatial omics sequencing by parallelization, miniaturization, and automatic methodological steps [15,16]. First, assay miniaturization in microfluidics improves enzyme reaction activity and reduces assay costs significantly. While traditional high-throughput single-cell analysis requires a reaction system of at least ~1 µL [17,18,19], each parallel reaction system in a microfluidic system ranges from a few picoliters to a few nanoliters, thereby improving the concentrations of reagents and reducing reagent consumption [20,21,22]. The water-in-oil system in Drop-seq is capable of generating approximately 3000 droplets per second with a volume of around 1 nL [23]. DroNc-seq, a modification of the Drop-seq method, can generate droplets with a volume of 300 pL at a higher rate (roughly 4500 droplets per second) [24]. Such improvement allows for scRNA-seq to be performed on cell nuclei. Second, parallel microchannels, microarrays, and microvalves can be used simultaneously for the precise manipulation of fluids to minimize batch effects. When combined with sequencing tools, massive parallel sequencing microdevices are capable of capturing and analyzing at the single-cell scale, which provides a chance for low-cost, high-throughput-based transcriptome analysis and opens up unprecedented opportunities to reveal cellular heterogeneity and its effects on cellular function. For example, Seq-well was able to profile thousands of primary human macrophages in a polydimethylsiloxane (PDMS) chip consisting of ~86,000 subnanoliter wells [21]. Similarly, when used for spatial transcriptome technology, microdevices allow for yielding spatial barcodes unbiasedly through parallel microchannels, resulting in thousands of different spatial spots [25]. Third, a single microfluidic chip enables the integration of multiple operations and compatibility with downstream measuring techniques. With the development of micro/nano manufacturing technology, experimenters can design and fabricate customized microfluidic chips for different clinical research applications. Collectively, as efficient, high-throughput integrated platforms, microfluidics can be applied to SCS and spatial omics sequencing to reveal the diversity of cell types in highly heterogeneous tissues, with further broad applications in developmental, neurological disease research, and more [26,27].

In this review, we survey the current landscape of microfluidic methods in the field of single-cell and spatial multi-omics and their operational principles, as well as assessing their relative advantages and limitations. Additionally, the applications of microfluidic-based methods in a wide range of fields including neuroscience, developmental biology, cancer, and more will be addressed. Last, we discuss future opportunities in the field of translational and clinical medicine.

## 2. Advances in Single-Cell RNA Sequencing Technologies

Traditional bulk sequencing methods obtain the average data for the gene expression profiles of cell populations [28,29], resulting in the consequent concealment of information at the single-cell level, such as molecular distributions and drug–target interactions. Thus, to understand the single-cell different expression (SCDE), new tools need to be developed to advance single-cell sorting and analysis. In 2009, Tang et al. published the pioneering work of single-cell genome-wide mRNA sequencing (scRNA-seq) [30], thereafter, the field exploded and received great attention (Table 1). Specifically, a significant increase in the number of cell types that can be processed with the throughput has increased from a few to several thousand.

SCS technologies can be distinguished as being either plate-based methods or microfluidic-based methods. Plate-based methods [19], represented by Smart-seq2 [17], are capable of obtaining full-length transcripts. However, the lack of suitable cell segregation strategies results in the quantity of cells available for analysis limiting the throughput of such methods [31]. Compared to conventional plate-based methods, the probability of sample cross-contamination can be reduced with microfluidic-based scRNA-seq methods, based on both microfluidics [20,23] and high-density microwells [21,32], resulting from the cell suspension being partitioned into nanoliter reaction chambers [20,21,22]. Importantly, this enables microfluidic platforms to be tailored to be compatible with different cell sizes [33,34], providing an expandable framework for a wide range of biological samples [20]. Given the parallelization, massive parallel omics sequencing, adopting microfluidics as a platform, provides a better performance in terms of consistency and throughput [21,35,36].

**Table 1:** Summary of Mmcrofluidic-based scRNA-seq technologies.

Technology	Year	Method	Sample Type	Fit for Rare Cell (Y/N)	Resolution	Ref.
Droplet-based scRNA-seq	2015	Drop-seq	Cell suspension	N	Single cell	[23]
Droplet-based scRNA-seq	2015	inDrop	Cell suspension	N	Single cell	[20]
Droplet-based scRNA-seq	2017	Chromium 10X	Cell suspension	N	Single cell	[37]
Droplet-based scRNA-seq	2017	DropNc-seq	Cell suspension	N	Single cell	[24]
Droplet-based scRNA-seq	2023	SPEAC-seq	Cell suspension	N	Single cell	[38]
Microwell-based scRNA-seq	2015	Cytoseq	Cell suspension	N	Single cell	[39]
Microwell-based scRNA-seq	2017	BD Rhapsody	Cell suspension	N	Single cell	[40]
Microwell-based scRNA-seq	2015	Solid-phase RNA capture	Cell suspension	N	Single cell	[36]
Microwell-based scRNA-seq	2017	Seq-Well	Cell suspension	N	Single cell	[21]
Microwell-based scRNA-seq	2020	Seq-Well S^3^	Cell suspension	N	Single cell	[41]
Microwell-based scRNA-seq	2018	scFTD-seq	Cell suspension	N	Single cell	[42]
Valve-based scRNA-seq	2014	Microfluidic Single-cell whole-transcriptome Sequencing	Cell suspension	N	Single cell	[22]
Valve-based scRNA-seq	2019	Hydro-Seq	blood	Y	Single cell	[43]
Valve-based scRNA-seq	2020	Paired-seq	Cell suspension	N	Single cell	[44]

### 2.1. Droplet-Based scRNA-Seq Methods

In droplet microfluidics systems, the cell suspension is partitioned into surfactant-stabilized droplets, followed by oil–water interactions to individually encapsulate aqueous droplets in an inert carrier oil. So far, all droplet-based scRNA-seq protocols can be categorized into the following five steps: the enrichment and assessment of target cells, encapsulation in droplets, reverse transcription (RT) and molecular amplification, sequencing, and data processing. The droplet microfluidic device greatly facilitates the sequencing throughput by the enrichment and encapsulation of live target cells. Moreover, the ability to naturally adapt to subsequent manipulations can provide a scalable framework for resolving single-cell information.

**Figure 1:**
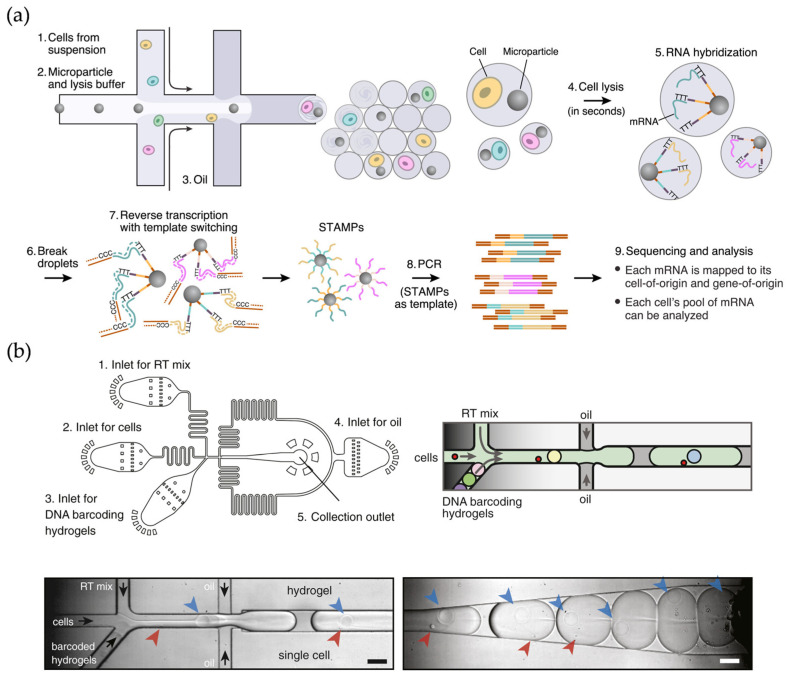
(**a**) Schematic diagram of the workflow of the Drop-seq technique. Reproduced with permission from Ref. [23]. Single cells are encapsulated in tiny droplets along with uniquely barcoded primer beads, enabling large-scale, highly parallel analysis. (**b**) Microfluidic device used in inDrop technology. Reproduced with permission from Ref. [20]. Cells are captured and barcoded in nanoliter droplets with high capture efficiency. Arrows indicate cells (red), hydrogels (blue), and flow direction (black).

Drop-seq [23] and inDrop [20], both published in 2015, are two representative works based on 3′ poly-A capture methods (Figure 1a,b). The two protocols use the same principle in terms of barcoding transcripts, but differ in other steps. Specifically, Drop-seq uses a non-deformable rigid resin for encapsulation, which means that both cells and beads obey the Poisson distribution, resulting in a low encapsulation efficiency. After the encapsulation, RT is performed in unison by pooling beads, which synthesizes barcoded primers directly for RNA capture. As opposed to Drop-seq, inDrop uses a droplet microfluidic device to generate barcoded hydrogel microspheres (BHMs). After the encapsulation, RT reactions occur in droplets individually, so the microreactors reduce reaction competition while bulk RT reactions reduce the batch effect, which is particularly important in high-throughput sequencing with a high assay precision. Importantly, Drop-seq is based on Smart-Seq [45] utilizing PCR-based template switching amplification, while inDrop is akin to CEL-Seq [46] following an in vitro transcription (IVT) protocol. Hence, being based on two different protocols means that both methods have their own strengths for amplification and DNA library preparation. Drop-seq has a much higher gene detection ability than inDrop in terms of sensitivity, especially for low-abundance transcripts. However, there is a significant bias in the quantitative analysis of RNA expression in the Drop-seq protocol due to PCR-induced non-linear amplification. On the contrary, CEL-Seq can greatly reduce amplification bias by barcoding and pooling samples before linearly amplifying mRNA with the use of one round of IVT. It is worth noting that the mRNA capture of both Smart-Seq and CEL-Seq requires poly (T) primers, resulting in important transcripts such as circRNAs that are without poly (A) tails being filtered out. Chromium 10X is a commercially available platform for scRNA-seq that delivers standardized capture conditions [37]. With a multichannel microfluidic chip, thousands of cells can be encapsulated into ~100,000 gel beads in emulsions (GEMs) in six minutes, resulting in single-cell capture efficiencies between 50% and 65%, with the probability of a single droplet capturing multiple cells being as low as 0.9%/1000 cells. Notably, such a short treatment time and reproducibility can reduce the probability of cell lysis and RNA degradation [37].

Based on the Drop-seq methodology, DropNc-seq achieves a higher capture rate efficiency and further obtains reliable nuclear expression profiles from frozen samples by combining with sNuc-Seq, a low-throughput method utilizing isolated nuclei [24]. In 2023, Wheeler et al. published SPEAC-seq, a forward genetic screening platform [38]. Two-cell encapsulated droplets achieved by microfluidics were co-cultured and then sorted through sequentially gating drops to obtain droplets encapsulating the desired cell–cell pairs, namely activated reporter cells and perturbed cells.

Overall, droplet-based scRNA-seq is severely affected by “doublets”, that is, one or more beads and/or cells in a droplet, which occurs for the following two reasons: 1. The presence of two-cell clusters in the suspension resulting from cell–cell interactions. 2. The encapsulation of beads and cells is random, and at least one of them is bound to be governed by the Poisson distribution, even if the variable beads are able to break the Poisson statistics in the case of a tight arrangement [47,48].

### 2.2. Microwell-Based scRNA-Seq Methods

In comparison to droplet-based scRNA-seq methods, microdevices based on microwell arrays can provide unbiased analysis in SCS at the ensemble level. Specifically, exactly the same nanoliter or picoliter microwells by the tens of thousands are used as stand-alone reactors and can load single cells of comparable sizes by gravity alone. Subsequently, cell lysis, RNA capture, and RT are performed in the original wells [21,39].

**Figure 2:**
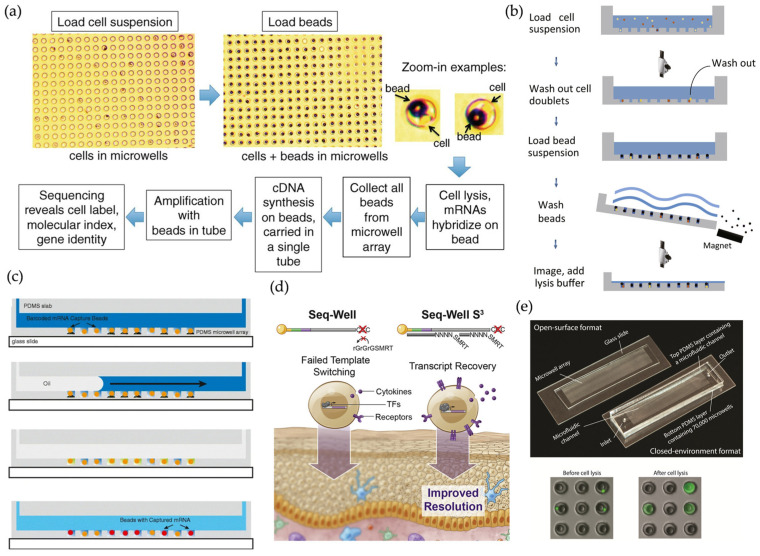
(**a**) Schematic and imaging data for CytoSeq. Reproduced with permission from Ref. [39]. The platform enables routine, digitized gene expression profiling of thousands of single-cell genes. (**b**) Experimental procedure for Microwell-Seq. Reproduced with permission from Ref. [32]. A high-throughput, low-cost platform capable of constructing mouse cellular maps at the single-cell level. (**c**) A schematic of the basic workflow for single-cell RNA printing. Reproduced with permission from Ref. [36]. Microwells in this platform enable low-cost printing of RNA on glass or capturing of RNA on beads. (**d**) Comparison between Seq-Well and Seq-Well S^3^. Reproduced with permission from Ref. [41]. Based on Seq-Well, Seq-Well S^3^ improves transcript capture and sensitivity. (**e**) scFTD-seq platform and fluorescence imaging data. Reproduced with permission from Ref. [42]. Portable and easy-to-use devices, as well as simplified and modular workflows facilitate clinical use.

In 2015, Fan et al. reported on Cytoseq, initially combining next-generation sequencing (NGS) with the stochastic barcoding of single cells via micropore arrays [39] (Figure 2a). In this strategy, to saturate all wells, the cell suspension concentration and the size of barcoded beads are informed by the geometry and dimensions of the wells. In 2018, Han et al. published a simple method named microwell-seq [32], which can lead to substantial reductions in labor and cost due to the reusability of the PDMS mold, coupled with the low cost of the agarose microwell arrays. Using the microwell arrays, the group characterized cellular heterogeneity and the cross-tissue cellular network with a minimal batch effect, as well as further publishing the first mammalian atlas [32] (Figure 2b). Moreover, given the operations, costs, and turnaround times, researchers may consider BD Rhapsody [40], a proven commercially available platform widely used in immune research and brain sciences [49,50], to observe cell-related information and implement integrative transcriptome and proteome analyses.

To reduce the risk of cross-contamination resulting from the open-top microwell design, the currently preferred method is to seal the microwell array. Bose et al. published a scalable platform for solid-phase RNA capture, consisting of printing RNAs on glass and capturing RNAs with polymer beads [36] (Figure 2c). After loading the cells into the wells and adding lysis buffer, there are different sealing protocols for different modes. The microwell array can be reversibly sealed by a mechanical sealer in the RNA printing mode and by oil injection in the RNA capture mode, respectively. The combination of solid-phase RNA capture with microarrays allows for the exchange of reagents without the need for altering the location of the captured material, which facilitates scalability and miniaturization by eliminating the need for multiple chambers. However, limited by the scale of the bead library and the size of the microwell array, the above platform has a lower throughput compared to Cytoseq. In another report, a portable platform named Seq-Well was developed by Gierahn et al. [21]. By the means of semipermeable membranes and surface-functionalized PDMS arrays, rapid solution exchange and reduced contamination across intercellular mRNAs can be achieved. Also worth mentioning is that Seq-Well uses the same library construction method as Drop-seq, where full-length transcripts are obtained by template switching after RT. However, this method requires a secondary PCR, which may result in the loss of validated products that are not tagged with the handle [51]. Hence, an iterative platform, Seq-Well S^3^, was reported in 2020, improving the library construction efficiency and gene detection sensitivity by using a randomly primed second-strand synthesis [41] (Figure 2d). scFTD-seq, published in 2018, proved that freeze–thaw was completely compatible with scRNA-seq [42] (Figure 2e). After the chip was filled with freeze–thaw lysis buffer, the microwell array was sealed using a glass slide, followed by three freeze–thaw cycles and room-temperature incubation. scFTD-seq reduced the cross-contamination, driven, in part, by the seal operation and lack of buffer exchange.

In conclusion, microwell-based scRNA-seq methods are theoretically easy to operate and cost-effective due to their fewer complicated peripherals and reagents. However, they are relatively less adaptable to rare cell analysis resulting from the fixed well sizes. Additionally, optimization strategies are still needed to effectively avoid cross-contamination.

### 2.3. Valve-Based scRNA-Seq Methods

Valve-based microfluidic chips manipulate the fluid flow behavior through pressure-controlled valves [22,43,44]. Specifically, the chip mainly integrates flow channels used for cell suspension flow, valve-controlled channels for changing the pressure, and elastically deformable PDMS films as valves. Changes in pressure control the state of the valve, thus, single-cell isolation can be further realized [52].

**Figure 3:**
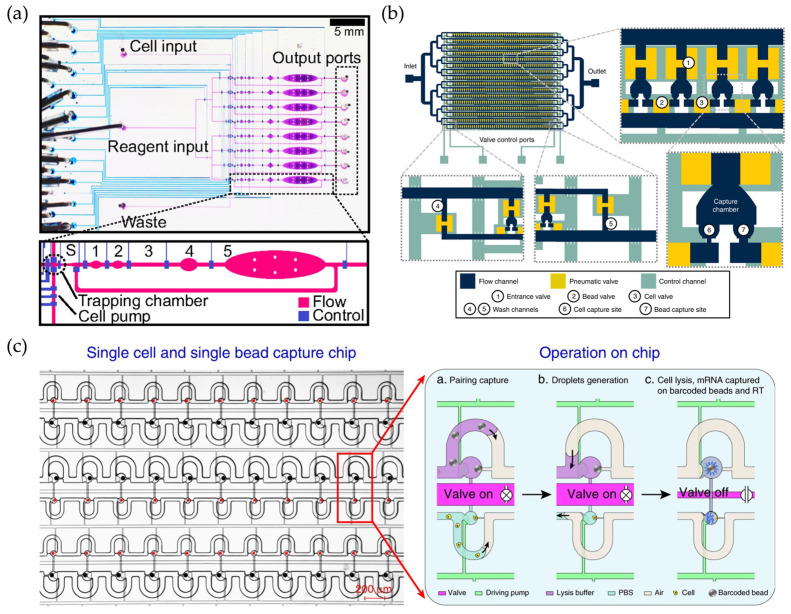
(**a**) Micrograph of the microfluidic device. Reproduced with permission from Ref. [22]. Injection of single-cell suspensions and reagents, and recovery of double-stranded cDNA from the output for single-cell whole transcriptome analysis. Lines indicate the control channels (blue), and the flow channels (purple). (**b**) Schematic diagram of Hydro-Seq technology. Reproduced with permission from Ref. [43]. The platform is capable of cleaning contaminants such as erythrocytes and could be used to isolate rare cells. (**c**) Design of Paired-seq chip. Reproduced with permission from Ref. [44]. The device is capable of efficiently isolating and removing cell-free mRNAs.

In 2014, Streets et al. proposed a semiautomated single-cell high-throughput sequencing program with a high sensitivity and precision [22] (Figure 3a). Individual cells from the cell suspension were sequentially directed by a peristaltic pump into each of the eight trapping chambers and pressurized into the sorting chamber, while multiple cells were discarded through the isolation valve. After sorting, the reagents were filled through the reagent input line and then the reactant was diffused and mixed by controlling the on–off state of the valve above the two chambers. Notably, the integration of valves and pumps had a significant advantage over well-based methods in terms of raw sample contamination removal, which makes it extremely suitable for scarce or easily contaminated samples, such as circulating tumor cells (CTCs). In 2019, Cheng et al. engineered Hydro-Seq chips that could process 10 ml of patient blood containing CTCs [43] (Figure 3b). The customized channel size and valve design captured individual CTCs from the sample into each chamber, while contaminants such as erythrocytes and leukocytes could be transferred. The difference in shape between the bead and capture ports facilitated bead loading. In addition, the valves were closed to the separated chambers, thus allowing for cell lysis and mRNA capture in the separated chambers.

In addition, the decontamination capability of the valve-based chip was also demonstrated in the removal of cell-free RNA. Zhang et al. introduced the Paired-seq platform, in which from hundreds to thousands of units were integrated for processing precious samples [44] (Figure 3c). Blocking valves and hydrodynamic differential flow resistance-based units had an immediate effect on the isolation and removal of cell-free mRNAs, which was further validated by subsequent sequencing.

The high-throughput systems above retain fragments of mRNA 3′ end information, while Fluidigm C1 [53], a commercialized platform, employs different protocols, including Smart-Seq [45] and CEL-Seq [46], to generate full-length transcripts. This standardized process reduces the requirements for experimental operations and, therefore, performs robustly and reproducibly in the characterization and data integration of clinical tissues [54] or blood samples [55]. Two prominent examples are the revealing of the intratumoral heterogeneity of the breast cancer transcriptome [54], as well as that of the cellular regulatory pathways in T-cell leukemia [55]. Nevertheless, this calls for caution regarding precious samples and samples that require high-quality gene expression data due to the low cell utilization and the risk of cell damage during the cell capture process [53].

Taking this into account, the valve-based chip structure enables multiple washes of cells and beads, thereby reducing the risk of sample contamination. It is worth noting that the manufacturing process for such SCS platforms, which is highly dependent on the chip structure, is relatively more complex. The integrated distribution of the chip, in particular the shape and design of the valves, greatly affects the efficiency and quality of single-cell capture. For example, the low Reynolds number in channels could be improved by integrating mixing valves, which results in an improved mixing efficiency during DNA amplification [56].

### 2.4. Single-Cell Multi-Omics Profiling

The central dogma of biology, which tightly links molecular species transcripts to DNA and proteins, implies that single omics information is insufficient to determine the biological function of eukaryotic cells [57,58]. On the one hand, the post-transcriptional status of genes varies from cell to cell within the same organism, or even the tissue, mainly depending on translation and degradation regulatory mechanisms. On the other hand, even in the same cell, the gene expression level fluctuates with time and external stimuli. Therefore, a single-cell multi-omics dataset can capture the static distribution and dynamic information inside and outside a cell to further analyze the mechanisms of gene expression and degradation regulation [59,60]. By mapping cell specificity and tracing cell lineages, the mechanisms of development and disease can be then elucidated [61].

The quantification of proteins as effector molecules of cellular function enables more accurate dynamic modeling to predict gene products and responses to perturbation [62,63]. As a platform capable of precise manipulation in microsystems, the microfluidic device has become a preferred system to integrate multiple quantification technologies at the single-cell level [64,65,66]. Initially, protein information is read out by the means of fluorescent signals. The relative maturity of imaging technology and analytic software affords immunofluorescence imaging a great potential for directly profiling protein levels [67]. Park et al. used microwell arrays with landmarks to isolate and localize single cells, so that the fluorescence intensity per well was able to quantitatively analyze protein expression dynamics. After cell lysis, the dynamics in transcript levels were obtained by fluorescence intensity via on-chip RT-PCR [68].

However, the throughput and number of fluorescence signal read-outs are limited by spectral overlap and resolution. Therefore, the key to the simultaneous high-throughput detection of the transcriptome and proteome lies in the translation of mRNAs and proteins into readable and unbiased signals. CITE-seq [64] and REAP-seq [69] are both sequencing-based high-throughput methods capable of co-profiling the transcriptome and proteome. Each is represented the following classical barcode-antibody binding mechanisms: streptavidin–biotin interaction and amine chemistry, respectively. Therein, CITE-seq, a widely used tool, is critical for identifying subsets of diseased cells [70], revealing tissue heterogeneity at disease stages [71], and analyzing the mechanisms of immune checkpoint molecular regulations [72]. Such methods are costly due to the use of antibody labeling proteins. In contrast, Delley et al. used oligonucleotide barcode-labeled aptamers, which allowed for the low-cost simultaneous characterization of the transcriptome and intracellular proteins [73].

**Figure 4:**
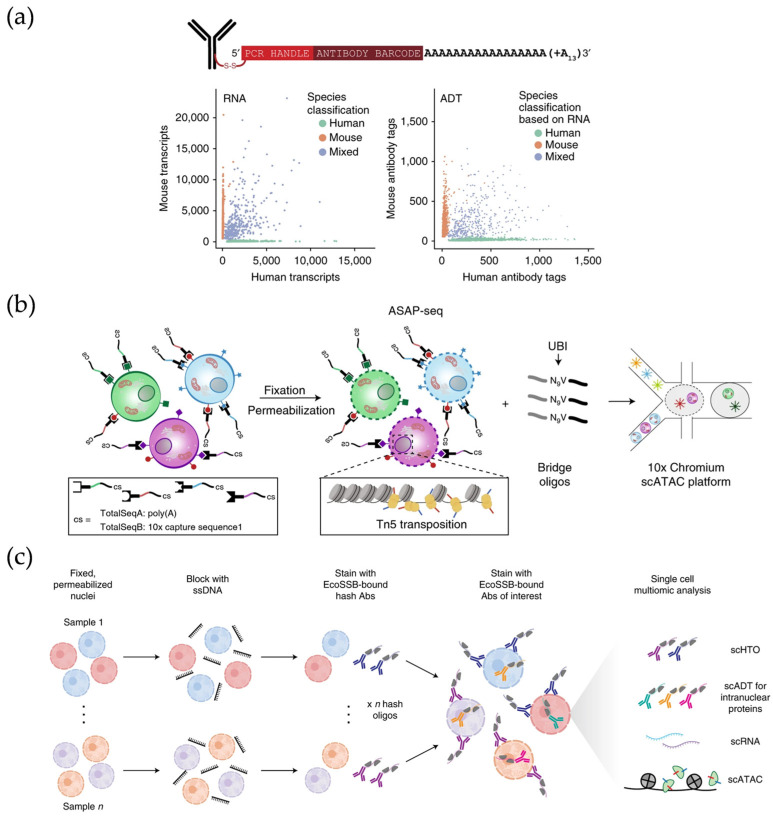
(**a**) Illustration of the DNA-barcoded antibodies used in CITE-seq and analysis of mixtures of mouse and human cells. Reproduced with permission from Ref. [64]. CITE-seq enables simultaneous detection of single cell transcriptomes and protein marks. (**b**) Schematic of ASAP-seq workflow. Reproduced with permission from Ref. [66]. ASAP-seq is capable of demonstrating chromatin accessibility on a genome-wide scale. (**c**) Schematic of NEAT-seq workflow. Reproduced with permission from Ref. [74]. NEAT-seq is a droplet-based microfluidics platform that can simultaneously analyze intracellular proteins, chromatin accessibility, as well as transcripts through intranuclear staining.

Another path to responding to genomic expression heterogeneity is to profile the genome and transcriptome in parallel. In 2014, Han et al. used a valve-based platform to isolate the same single-cell mRNA from gDNA. Subsequent RT as well as denaturation and neutralization were performed on the microarray, followed by almost synchronous amplification. Moreover, genes and gene products are not two-by-two linear pathways, and, thus, triple-omics and higher-order single-cell multi-omics are in the process of inferring genomic regulatory models in unprecedented detail [31]. In 2021, Mimitou et al. released ASAP-seq, which is capable of demonstrating chromatin accessibility on a genome-wide scale [66] (Figure 4a). Compatible with multimodal assays such as CITE-seq, ASAP-seq enables a triple- or four-order single-cell multi-omics workflow, revealing stimulus-dependent dynamic changes in T cells. In 2022, Chen et al. profiled CD4 memory T cells with NEAT-seq, a droplet-based microfluidics platform that can simultaneously analyze intracellular proteins, chromatin accessibility, and transcripts through intranuclear staining [74] (Figure 4b).

In conclusion, due to its precise and unbiased analysis, as well as the comprehensive validation provided on cellular functions and temporal dynamics, microfluidic-platform-based multi-omics sequencing technology is able to reveal the etiology of diseases and highlight disease indicators by integrating multi-omics data. Despite still being in its infancy, the capture of multiple layers provides a time-saving and efficient method for predicting, selecting, and validating regulatory factors of interest. The increased ability to analyze the heterogeneity originating from genetic and environmental stimuli facilitates the further interrogation of molecular regulatory networks spanning the central dogma.

### 2.5. Technical Challenges and Future Directions

In summary, the challenge in useable yields and data quality in SCS technologies is to reduce cross-contamination so that real single-cell information is obtained [75]. Specifically, each system should ideally (i.e., no cross-contamination) contain both one cell and one bead. However, subject to the Poisson distribution, there may be no or multiple cells and/or beads in the system. In particular, complex components in blood samples such as erythrocytes and cell-free RNA can further introduce additional contamination. Though informatics have been developed to work on this issue from an algorithmic perspective [76,77], microfluidic-based single-cell sequencing methods can minimize cross-contamination in the simplest way possible from a system design. For example, the close-packed arrangement of deformable beads can break the Poisson distribution [47], the sealing of microwell arrays can efficiently avoid inter-system contamination, and the integration of valves and pumps can efficiently scrub contaminants from samples.

## 3. Advances in Spatial Transcriptomics

While SCS methods provide an exhaustive account of intercellular heterogeneity, they fail to explain the interactions between cells and with the surrounding microenvironment, as well as the spatial distribution [9,78]. Spatially resolved transcriptomics (SRT) technologies have fundamentally reshaped researchers’ understanding of tissue homeostasis, tissue development, and even disease mechanisms. SRT technologies can be summarized into the following three categories: imaging-based technologies, laser capture microdissection (LCM)-based technologies, and sequencing-based technologies. Imaging-based technologies are limited by the limited number of fluorescent probes and the complexity of the coding process [79]. In LCM-based technologies, cutting a tissue section with a laser is costly and the analysis area is limited [80]. In contrast, sequencing-based technologies have the advantage of being unbiased, high-throughput, and producing low error [81,82].

Microfluidic systems load cellular information into space through unique array features or microchannels, and further quantify the mRNA expression of a large number of genes in the spatial dimension through high-throughput sequencing (Table 2). The exploration of spatial heterogeneity and the reconstruction of gene expression profiles have deepened the understanding of diseases, neurology, and cancer [83]. Of note, another important implication of SRT technology is to map developmentally relevant spatiotemporal transcriptomics, exploring the developmental trajectory of the embryo, as well as the evolution of life in time and space [84,85].

### 3.1. Array-Based SRT Methods

Array-based SRT methods have been widely used to obtain spatially resolved genetic information [9]. Broadly, polyadenylated RNA capture is divided into surface capture and microbead capture [87,88]. The surface capture immobilizes capturing probes by modifying chemical groups, such as epoxy groups, on the surface of glass or silicon wafers [87]. A higher spatial resolution can be achieved by increasing the density of the modification. The microbead capture attaches the capturing probes to microbeads, and the accuracy increases with a decreasing volume of microbeads [89].

**Figure 5:**
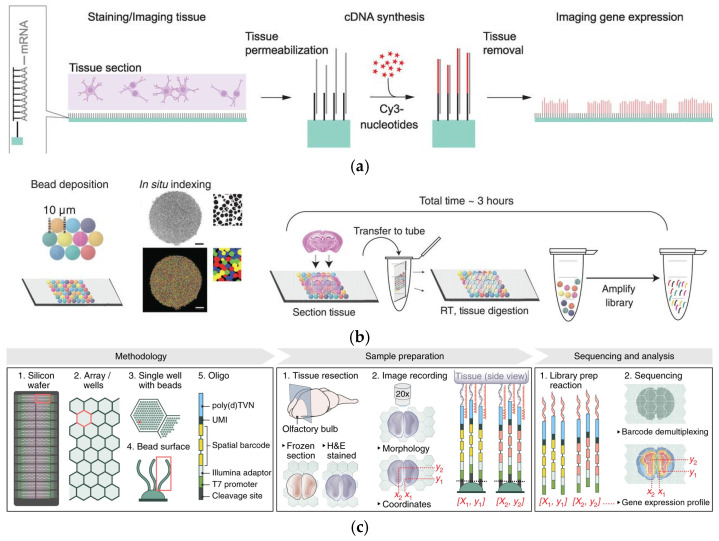
(**a**) Spatially localized cDNA synthesis. Reproduced with permission from Ref. [87]. ST was the first use of surface capture and commercialized as the Visium platform. (**b**) Slide-seq workflow. Reproduced with permission from Ref. [88]. Slide-Seq method deposited 10 μm magnetic beads on the plane of the slide to capture tissue mRNA. (**c**) Schematic diagram of HDST. Reproduced with permission from Ref. [89]. HDST method modified 2 μm magnetic beads in the microwells of the slide to capture tissue mRNA.

Spatial transcriptomics (ST), the first use of surface capture, was proposed by Stamathl et al. [87] and commercialized as the Visium platform (Figure 5a). In total, 1000 + trapping regions 200 µm apart were modified on the slide in ST. After RT of the mRNA from tissue sections captured in situ, cDNA–mRNA complexes with different spatial barcodes were used for library preparation and NGS readout. In the Visium platform, this distance was reduced to 100 μm to achieve a higher spatial resolution. Based on the above platforms, SM-Omics [89] and SPOTS [90] were able to analyze transcriptome information and a panel of protein information from the same tissue section on arrays with a 100 µm and 55 µm resolution, respectively. Slide-Seq technology [88] and HDST technology [89] are representative of microbead capture schemes (Figure 5b,c). Specifically, after modifying magnetic beads with spatially barcoded RT primers, the Slide-Seq technology deposited 10 μm magnetic beads on the plane of the slide, whereas the HDST technology modified 2 μm magnetic beads in the microwells of the slide to capture tissue mRNA. It is worth noting that, although the resolution can be close to that of a single cell, these beads do not capture efficiently enough to support the amount of data required for single-cell analyses. In addition, the spatial barcodes actually map back to adjacent tissue sections, which results in inaccuracy that is quite sensitive to highly heterogeneous samples such as tumors.

### 3.2. Microchannel-Based SRT Methods

**Figure 6:**
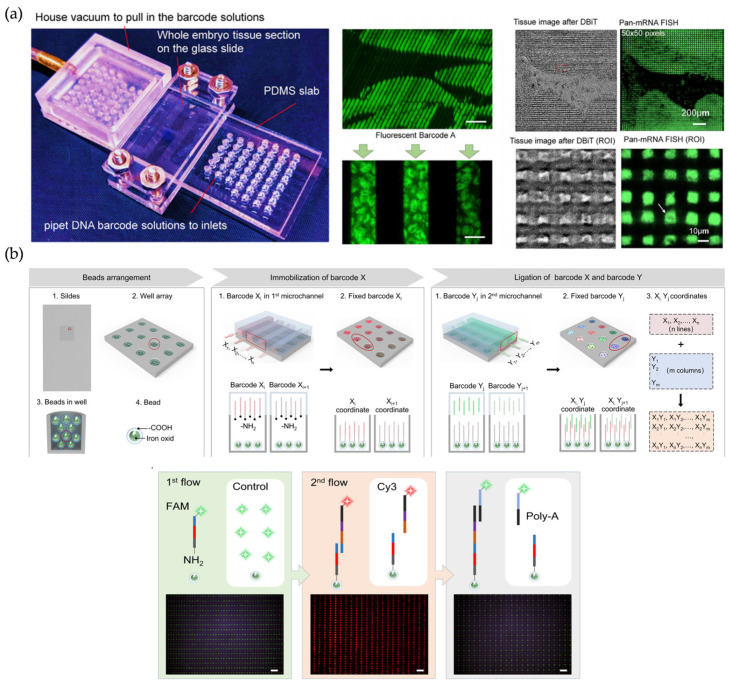
(**a**) Microfluidic device used in DBiT-seq. Reproduced with permission from Ref. [91]. DBiT-seq is capable of analyzing the proteome, transcriptome, and transcriptome of the same tissue section while maintaining a high resolution. (**b**) Schematic diagram and validation of Well-ST-Seq technology. Reproduced with permission from Ref. [25]. Well-ST-Seq avoids channel blockage problems associated with tissue deformation.

Microchannel-based SRT methods offer an unprecedented perspective on the analysis of biological regulatory networks due to their flexibly adjustable spatially barcoded pixels and their ability to integrate various omics layers. Distinguishing from the solid-phase matrix capture of biomolecules described above, DBiT-seq delivered barcodes for the direct localization of nucleic acids or proteins onto tissue sections [91] (Figure 6a). The key to the method was the sequential use of two PDMS microfluidic chips lined with 50 parallel microchannels. Since the microchannels between the two chips were oriented vertically, the intersecting pixels formed a spatial grid. The resolution of the above pixels could reach up to 10 μm and capture about 2000 genes by adjusting the distance between the microchannels. DBiT-seq is capable of analyzing the proteome, transcriptome, and transcriptome of the same tissue section while maintaining a high resolution [91]. It is also compatible with optical image characterization, which makes the chip capable of an all-round accurate analysis of multi-omics data based on morphological structure. In addition to FF sections, this strategy is also compatible with FFPE tissues with an accuracy of a 50 μm point spacing [92]. Furthermore, microchannel-based PDMS chips can also be used to modify paramagnetic particles, thus avoiding the channel blockage problems associated with tissue deformation while retaining the advantage of flexible resolution adjustment [25] (Figure 6b).

As a universal and compatible strategy, microchannel-based SRT methods represented by DBiT-seq extend [93,94] or integrate [95,96] multi-omics data such as proteomes, epigenomes, and so on, thus realizing multiple spatial co-analysis.

### 3.3. Spatiotemporal Transcriptome

Gene expression and cell-state transitions change continuously in organisms. In order to obtain dynamic multi-omics information, it is necessary to obtain dynamic expression datasets in the temporal and spatial dimensions. The spatiotemporal transcriptome is based on spatial transcriptome technology, which combines spatial information with pseudotime [97,98]. By identifying and tracking key genes during cell migration and differentiation, a deeper understanding of cell fate and processes such as embryonic development and neural differentiation in species including Mus musculus and Homo sapiens can be achieved [84,99]. The size and morphology of fresh-frozen sections under pseudotime are constantly changing with development [100]. Microfluidic devices are capable of adjusting the resolution and size to target regions of interest for comprehensive and detailed analyses [101]. In addition, the low cost and high throughput of microfluidics make it suitable for sequencing large quantities of embryos at different time points [102,103]. Unbiased technical strategies prefer to impute data with spatial and pseudo-temporal contexts as complete cellular differentiation axes. This implies that, when facing problems such as lineage bifurcation decisions, in situ hybridization-based spatiotemporal transcriptomes only target a limited number of target genes, while some capture-based spatiotemporal transcriptomes are unbiased under complex embryonic environments, such as during gastrulation [104].

**Figure 7:**
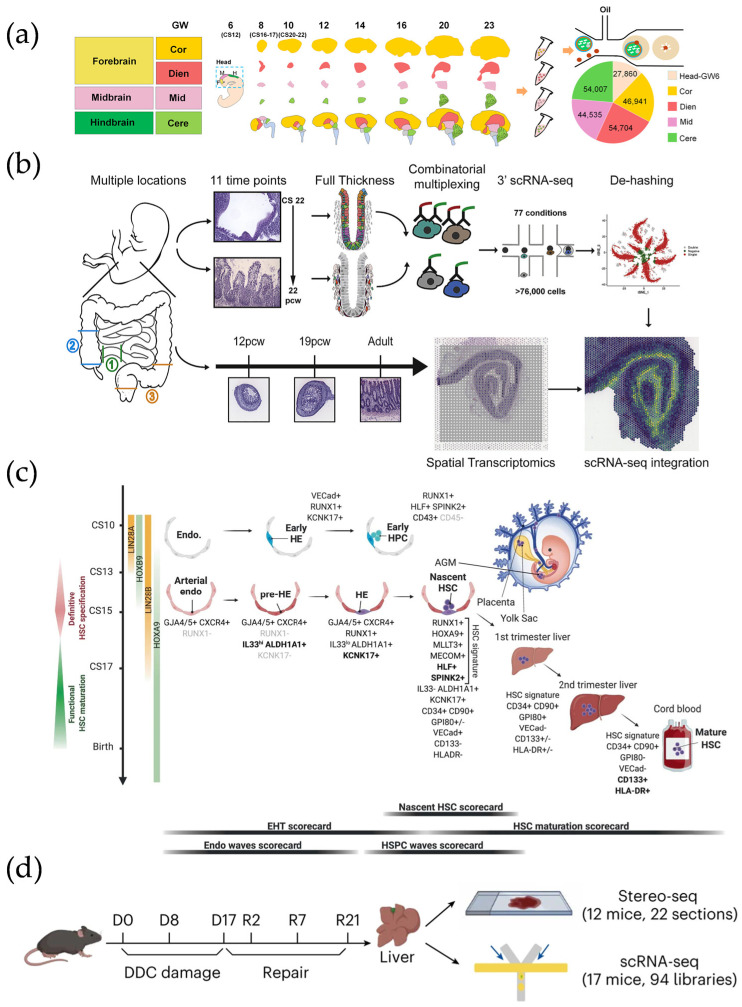
(**a**) Location, period, study design, and number of cells sequenced information. Reproduced with permission from Ref. [100]. (**b**) Overview of study design for intestinal development atlas. Reproduced with permission from Ref. [105]. (**c**) A schematic displaying the key cell types, stages, and markers involved in human hematopoietic stem and progenitor cell (HSPC) specification, emergence, and maturation. Reproduced with permission from Ref. [84]. (**d**) Schematic of DDC-induced injury and repair. Reproduced with permission from Ref. [109]. Spatiotemporal analyses of cholestatic injury and repair processes in mice reveal the potential of the spatiotemporal transcriptome for tissue injury and regeneration studies.

Higher orders of magnitude of sensitivity and capture efficiency are key to regionalizing spatiotemporal dynamics. To date, researchers have maintained a considerable interest in the development of the mammal post-implantation embryo at all periods of gestation [101,105,106,107]. Slide-seqV2 identified and recapitulated mouse neocortex developmental trajectories at a near-cellular resolution [80,108]. With nearly an order of magnitude higher sensitivity than the original Slide-seq, more than a thousand key genes during embryonic eye development and genetic drivers of disease could be identified. Subsequently, the spatiotemporal transcriptome has continued to increase in the field of view and resolution. For example, Stereo-seq (Figure 7a), with its cellular resolution and large field of view, was able to track the embryonic organ development trajectory and transcriptional variation in whole mouse embryos spanning E9.5–E16.5 [81], as well as reveal the widest spatiotemporal development of different regions of the human brain across time points to date [100]. sci-Space could elucidate the developmental orientation of nerves in three spatiotemporal dimensions with a single-cell precision by bringing a barcode to each nucleus [102].

In addition to whole mouse embryos, spatiotemporal transcriptome technology can characterize human organic morphogenesis by linking SCS and SRT. Fawkner-Corbett et al. depicted developmental events in full-thickness intestinal tissue and revealed time-critical transcriptional defects [105] (Figure 7b). Recently, as part of the Human Cell Atlas program, the characterization of the intertemporal development of human embryonic limb organs was made feasible [110]. The understanding of hematopoietic stem cells (HSCs) development has major implications for the diagnosis, prognosis, and treatment of hematologic diseases [111]. The combination of scRNA-seq and Visium commercial platforms elucidated the cellular origins of HSCs and confirmed the sites of HSC emergence and the transcription factor mechanisms. Establishing and visualizing a developmental roadmap for HSCs provides perspectives to guide in vitro transplantable HSC manufacturing efforts [84] (Figure 7c).

The spatiotemporal transcriptome can demonstrate the clinical relevance of developmental cellular mapping [112,113,114,115]. Spatiotemporal analyses of cholestatic injury and repair processes in mice reveal the potential of the spatiotemporal transcriptome for tissue injury and regeneration studies [109] (Figure 7d). By characterizing the gene expression for different disease periods, researchers are able to accurately assess the horizontal expression of specific genes during changes in organ morphology versus heterogeneity [116].

### 3.4. Technical Challenges and Future Directions

Overall, microfluidic-based SRT methods reduce the limitations of cost and laboratory equipment, obtaining thousands of capture spots with different spatial barcodes in an unbiased manner [110]. However, the system performance of such methods is limited by the process of microfluidic devices. First, there is a risk of extremely minute fluid exchange between the adjacent microchannels of the microfluidic device, which leads to unavoidable contamination during spatial barcode probe modification and tissue section encoding. Second, microfluidic-based SRT methods carry the risk of mRNA diffusion during permeabilization compared to SRT methods using hybridization probes. A promising approach is the domain restriction of tissue sections by microwell substrate processing. Finally, while other SRT techniques have achieved a nanometer resolution, the resolution of microfluidic spatial transcriptomic methods is limited to the micrometer level by lithography and nanostructure fabrication. Although efforts have been made to reach up to a 10 μm resolution [25,91], how to obtain spatial information at the subcellular level remains a future challenge [85,117].

## 4. Clinical Applications and Conclusions

SCS technologies and SRT technologies bridge the gap between clinical histopathology and molecular phenotyping [118], enabling the corroboration of experimentally obtained information on in situ intercellular molecular communication with clinical medical phenomena. When standardized and reproducible sequencing data are combined with clinical precision therapies, SCS technologies and SRT technologies will be able to assist in predicting potential therapeutic targets and even therapeutic effects, thus further advancing researchers’ understanding of developmental processes and disease models and reshaping precision therapeutic protocols [59,118,119,120].

### 4.1. Early Detection and Disease Classification in Oncology

In translational and clinical medicine, ‘one size fits all’ therapeutic approaches often result from a lack of the timely subcategorization of diseases and accurate prognoses [121]. In fact, microorganisms may activate a high expression of metabolic pathways [122]. The tumor microenvironment (TME) and highly heterogeneous tumor tissues form a complex immune network [120]. In the above ecosystem, multiple highly variable behaviors, such as immune cell infiltration and immune evasion, further influence the accurate judgment of tumor diagnosis and clinical treatment [123,124].

**Figure 8:**
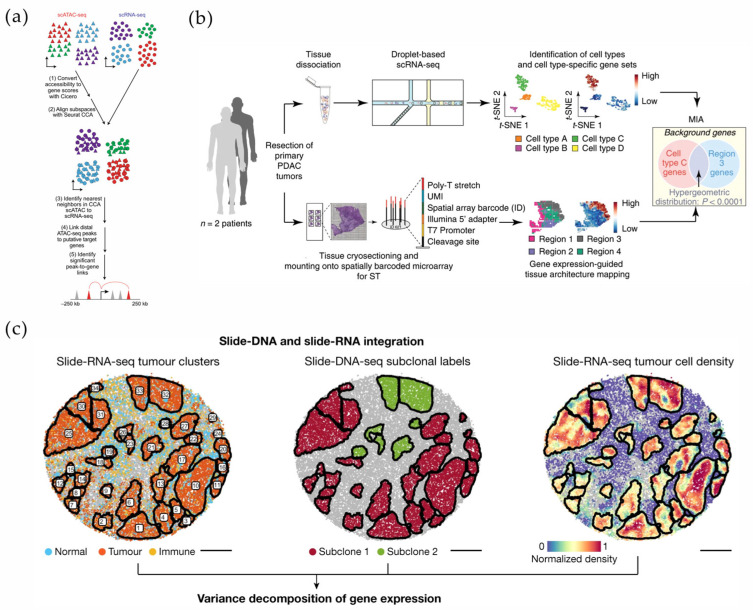
(**a**) Schematic for alignment of scATAC-seq and scRNA-seq data. Reproduced with permission from Ref. [128]. scATAC-seq has simultaneously tagged and fragmented DNA sequences, such as 10x Multiome, in open chromatin regions and further paved the way for other technologies that profile both the epigenome and transcriptome in single nuclei. (**b**) Schematic for alignment of snRNA-seq and Stereo-seq. Reproduced with permission from Ref. [129]. Multimodal intersection analysis (MIA) across the two datasets has revealed the spatial distribution of the cell populations and subpopulations. (**c**) Slide-DNA and slide-RNA integration. Reproduced with permission from Ref. [123]. Slide-DNA-seq has captured spatially resolved DNA sequences from intact tissue sections. The integration of slide-DNA-seq and spatial transcriptomics has uncovered distinct sets of genes that are associated with clone-specific genetics.

Spatial transcriptome technology tracks the cancer evolution present in cancerous regions and histologically benign tissues adjacent to cancer in an unsupervised manner, redefining distinct clonal patterns and cancer evolution with an unprecedented spatial resolution [125]. In addition, microfluidics and its expanded platforms have important implications for the deconstruction of hemato-oncological ecosystems in complex microenvironments, such as aberrant regulatory programs and inter-tumoral variability [126]. The results of SCS were able to classify spinal stromal cells and dissect their responses and molecular abnormalities under AML [127]. In a study of mixed-phenotype acute leukemias, single-cell multi-omics analysis inferred a transcription factor, RUNX1, that influences leukemia survival [128] (Figure 8a).

Currently, scRNA-seq and ST, either alone or in combination, have been developed as powerful tools for interpreting pathological phenomena and evaluating disease-specific markers in complex or highly heterogeneous tissues. In the process of disease diagnosis and targeted therapies, the information obtained through this emerging technology platform covers the scope and dimensions from cell-to-cell interactions to intracellular transcriptional mechanisms, from holistic networks to dynamic microenvironments, and from tissue heterogeneity to single-cell heterogeneity [16,61,125,130,131]. Multimodal intersection analysis (MIA) enables the integration of scRNA-seq and ST datasets from the same lesion region and the subsequent spatial assessment of the cell types enriched in the region or cluster by the degree of overlap of specifically expressed genes between the two. As an early mode of dataset integration, MIA provides spatial cell specificity, as well as an immune repertoire of lesion regions such as pancreatic ductal adenocarcinoma (PDAC) [129], esophageal squamous cell carcinoma [132], and cervical squamous carcinoma [133] (Figure 8b).

In conclusion, the high degree of tumor heterogeneity poses a great challenge for the prospective validation of precision therapeutic processes. The integration of high-resolution multi-omics analyses will be required to accelerate the analysis of complex biomarkers and therapeutic targets. For example, Slide-DNA-seq enables the spatial analysis of clonal heterogeneity and provides an avenue for analyzing copy number aberrations (CNAs), one of the drivers of cancer progression [123,131] (Figure 8c).

### 4.2. Cellular Dynamics of the Brain

The central nervous system (CNS) directs the complex activities and interactions of billions of neurons and glial cells [134]. Understanding the nuances between cells through SCS techniques plays a crucial role in the study of neurodevelopment and neurological diseases [135,136,137].

The highly dynamic brain is the key to the CNS, and analyzing the cerebrospinal fluid (CSF), which protects the CNS, can help in the diagnosis of CNS diseases. Single-cell transcriptomics has been able to determine the cellular profile of cerebrospinal fluid versus blood in an unbiased manner, increasing the diversity of the cellular composition of the CSF and the transcriptional phenotypes of the cells in the bloodstream [138]. In the study of Alzheimer’s disease (AD), the integration of snRNA-seq and snATAC-seq datasets is able to identify the transcription factors (TFs) for changes in disease gene expression at a single-nucleus resolution [139]. Furthermore, in addition to exploring disease mechanisms and screening for therapeutic targets, the combination of peptide screening has enabled further analyses of adaptive immune responses in the CSF and blood of patients [99,140].

The connectome, as the key to the functioning of the nervous system, is used to refer to all elements and connections [141]. In animal models, studies of the brain and spinal cord have enriched cell population types and elucidated cell-type-specific responses [103]. Currently, work has been conducted to identify and map cell types with the help of a combination of SCS, SRT, and retrograde labeling. Brain-wide atlases of gene expression constructed by such work reveal the complex topology of the brain connectome and further bridge the gap between the transcriptome and connectome [142]. The Allen Mouse Brain Atlas (AMBA) [143,144] and the Allen Human Brain Atlas (AHBA) [145], the most widely used brain atlases, quantify tens of thousands of genes in the brain. Such comprehensive mapping of the molecular characterization and the spatial patterns of gene expression will help to extend our mechanistic studies of neurodevelopmental and neurodegenerative diseases [142].

### 4.3. Discussion and Future Directions

Cellular heterogeneity among tissues poses significant challenges for molecular mechanisms and pathology analyses [146,147]. The controllable strategies of microfluidic devices provide new horizons for single-cell, spatial, and spatiotemporal histology. For example, the high-precision manipulation of single cells for SCS [20,21,32] and reliably and reproducibly unbiased capture for SRT [87,88,91] make microfluidics the most promising high-throughput platform. With cost and efficiency in mind, a key question is undoubtedly how researchers can match the most appropriate sequencing method, whether SCS or SRT, for existing biological samples to accurately detect and quantify biomolecules.

First, the sample type plays a decisive role in the selection of sequencing methods. Highly heterogeneous samples such as tumors require deeper and more precise scRNA-seq methods to reveal signaling pathways and stemness, etc. [148]. The introduction of microfluidics has led to a great improvement in the throughput and precision of scRNA-seq compared to traditional tubes and plate-based sequencing. On this basis, more and more characterization modalities are focusing on clinical applications and presenting unprecedented opportunities for precision therapy [149]. A current challenge is that difficult-to-access disease tissues limit sample types and sample sizes. In this context, stem-cell-derived organoids are a highly promising option [150].

Second, a corollary trend is the characterization of comprehensive molecular phenotypes at the single-cell level [151,152]. While single histologic data can provide predictable information with disease progression, for complex diseases such as type 2 diabetes (T2D), osteoarthritis, etc., the combination of transcriptomics, proteomics, metabolomics, etc., can refine the molecular mechanisms of disease etiology, which can then be used for prediction and diagnosis in the clinic [153,154,155,156,157]. Therefore, how to integrate information across these molecular layers is a key issue. Under this premise, a promising direction is the processing of sequencing data [158]. In the process of biomolecular analysis, it is an inevitable trend to go from the whole to the details and from the details back to the whole. To date, the preferred probabilistic approach for revealing complex organizational structures has been the back-convolution of single-cell data against specified spatial transcriptome data. In addition, for example, one of the effective ways to improve spatial annotation is to superimpose expression signatures on single-nucleus RNA-sequencing data [159]. In addition, identifying and removing low-quality sequencing data is the key to obtaining robust multi-omics analysis results [160]. For microfluidic-based SCS methods, the technical noise/dropout events and the methodological limitations of using single-end sequencing largely affect downstream analysis. In this context, imputation is a useful strategy to replace the missing data, thereby restoring the true expression level of the genes [161,162]. For microfluidic-based SRT methods, the tissue morphology, physical distance between data points, and gene expression need to be considered simultaneously. For example, for both healthy and diseased tissues, Pham et al. developed a spatial graph-based method to robustly interrogate biological processes [163].

Finally, in translational research, solid tumors and CTCs have garnered a high interest in the field of single tumor cell research [121,164]. Analyses targeting highly heterogeneous tumor cells have benefited from the application of highly parallel microfluidic devices. The combination of transcriptomics and proteomics has proven to be a highly successful approach to interrogating complex disease mechanisms [165,166]. It is foreseen that the integration of multi-omics information at a single-cell resolution will greatly provide life science and modern medicine, with the capability for early disease prediction and targeted precision therapy.

## Figures and Tables

**Table 2:** Summary of microfluidic-based spatial transcriptomics technologies.

Technology	Year	Method	Sample Type	Array Substrate	Resolution	Ref.
Array-based SRT	2016	ST	FF	Glass	100 μm	[79]
Array-based SRT	2019	Visium	FF	Glass	55 μm	[86]
Array-based SRT	2019	Slide-Seq	FF	Glass	10 μm	[81]
Array-based SRT	2019	HDST	FF	Silicon	2 μm	[79]
Microchannel-based SRT	2020	DBiT-seq	FF/FFPE	Microfluidic Channel divided tissue	10–50 μm	[82,83]
Microchannel-based SRT	2023	Well-ST-seq	FF	PDMS	10–50 μm	[25]

FF: Fresh-frozen section. FFPE: Formalin-fixed paraffin-embedded section.

## Data Availability

Not applicable.
